# Joint hypermobility in athletes is associated with shoulder injuries: a systematic review and meta-analysis

**DOI:** 10.1186/s12891-021-04249-x

**Published:** 2021-04-26

**Authors:** Behnam Liaghat, Julie Rønne Pedersen, James J. Young, Jonas Bloch Thorlund, Birgit Juul-Kristensen, Carsten Bogh Juhl

**Affiliations:** 1 Department of Sports Science and Clinical Biomechanics, University of Southern Denmark, Campusvej 55, 5230 Odense M, Odense, Denmark; 2 Department of Research, Canadian Memorial Chiropractic College, Toronto, Canada; 3 Research Unit for General Practice, Department of Public Health, University of Southern Denmark, Odense, Denmark; 4 Department of Physiotherapy and Occupational Therapy, Copenhagen University Hospital, Herlev and Gentofte, Copenhagen, Denmark

**Keywords:** Joint instability, Sports, Shoulder injuries, Meta-analysis, Risk factors

## Abstract

**Background:**

Joint hypermobility in athletes is associated with increased risk of knee injuries, but its role in relation to shoulder injuries has not been scrutinized. Therefore, our aim was to synthesize the evidence on the association between joint hypermobility and shoulder injuries in athletes.

**Methods:**

Data sources were MEDLINE, CINAHL, EMBASE, and SPORTDiscus from inception to 27th February 2021. Eligibility criteria were observational studies of athletes (including military personnel), mean age ≥ 16 years, and with a transparent grouping of those with and without joint hypermobility. A broad definition of joint hypermobility as the exposure was accepted (i.e., generalised joint hypermobility (GJH), shoulder joint hypermobility including joint instability). Shoulder injuries included acute and overuse injuries, and self-reported pain was accepted as a proxy for shoulder injuries. The Odds Ratios (OR) for having shoulder injuries in exposed compared with non-exposed athletes were estimated using a random effects meta-analysis. Subgroup analyses were performed to explore the effect of sex, activity type, sports level, study type, risk of bias, and exposure definition. Risk of bias and the overall quality of evidence were assessed using, respectively, the Newcastle–Ottawa Scale and the Grading of Recommendations Assessment, Development and Evaluation (GRADE).

**Results:**

Among 6207 records, six studies were included with 2335 (range 118–718) participants (34.1% females; athlete mean age 19.9 years). Athletes with joint hypermobility were more likely to have shoulder injuries compared with athletes without joint hypermobility (OR = 3.25, 95% CI 1.64, 6.43, I^2^ = 75.3%; *p* = 0.001). Exposure definition (GJH, OR = 1.97, 95% CI 1.32, 2.94; shoulder joint hypermobility, OR = 8.23, 95% CI 3.63, 18.66; *p* = 0.002) and risk of bias (low, OR = 5.25, 95% CI 2.56, 10.8; high, OR = 1.6, 95% CI 0.78, 3.29; *p* = 0.024) had large impacts on estimates, while the remaining subgroup analyses showed no differences. The overall quality of evidence was low.

**Conclusion:**

Joint hypermobility in athletes is associated with a threefold higher odds of having shoulder injuries, highlighting the need for prevention strategies in this population. However, due to low quality of evidence, future research will likely change the estimated strength of the association.

**Protocol registration:**

Open Science Framework registration osf.io/3wrn9.

**Supplementary Information:**

The online version contains supplementary material available at 10.1186/s12891-021-04249-x.

## Introduction

Sports-related traumatic shoulder injury and shoulder pain are frequently reported in athletes [1–3]. Besides pain, emotional distress, and interrupted sports participation, athletes can experience reduced work capacity, in addition to impaired shoulder-related quality of life [4, 5]. The incidence of shoulder injuries in overhead sports reaches up to 3.3 per 1000 athlete exposure hours, [6–8] and incidence of shoulder dislocations have been reported to range between 0.12 (practice injuries) and 0.31 (game injuries) per 1000 athlete exposure hours [9], with a higher incidence rate in young males compared with older males and females in general [10, 11]. There is an increased risk of recurrent and subsequently chronic instability following a primary shoulder dislocation [12–14]. Therefore, identifying risk factors and developing strategies to prevent shoulder dislocation and subsequent instability are key components of shoulder injury prevention [15].

Joint hypermobility, characterised by an ability to move the joints beyond the normal range of motion, considering the age, sex, and ethnic background of the individual, [16–18] can potentially be a risk factor of shoulder injuries. Various case definitions have been described, including inherent joint hypermobility, acquired laxity following a traumatic injury, and hypermobility due to an adaptation to a specific sport and/or physical activity. Further, joint hypermobility can be seen in relation to a single joint or as generalised joint hypermobility (GJH) affecting several joints. A common feature of GJH is the presence of pain, dislocations, subluxations, and joint sprains [19–21]. During sports activities, localised joint hypermobility and GJH have been reported to increase the risk of sustaining an injury [22–24]. While previous reviews have found an association between joint hypermobility and an increased risk of knee injuries, including anterior cruciate ligament injuries, [25–27] the role of joint hypermobility in relation to shoulder injuries has not been scrutinized. Therefore, our aim was to synthesize the evidence on the association between joint hypermobility and shoulder injuries in athletes. Our main hypothesis was that joint hypermobility increases the risk of sustaining a shoulder injury.

## Methods

This systematic review was performed in accordance with the guidelines from Cochrane [28] and reported according to the Preferred Reporting Items for Systematic Reviews and Meta-analysis (PRISMA) guidelines [29]. The study protocol was pre-registered and made publicly available at Open Science Framework registration osf.io/3wrn9.

### Eligibility criteria

This paper included cohort studies, cross-sectional studies, and case-control studies, published in full-text in English, assessing the association between joint hypermobility and shoulder injuries. Conference abstracts were excluded.

The population of interest was athletes (including military personnel) with a mean age ≥ 16 years participating in any type and level of sport or military activities. Studies were excluded if their participants had systemic inflammatory rheumatic diseases, connective tissue diseases (Marfan syndrome, Stickler syndrome, Loeys-Dietz syndrome, Ehlers-Danlos Syndrome), or neurological diseases.

For exposure, a broad definition of joint hypermobility was accepted (i.e., generalised joint hypermobility (GJH) and shoulder joint hypermobility). Since there is no consensus about the exact definition of shoulder joint hypermobility, shoulder instability and laxity were accepted as relevant definitions, without excluding studies based on their measurement tool or threshold, nor differentiating between inherent or acquired joint hypermobility. Studies were excluded if they did not use a threshold to distinguish between participants with or without joint hypermobility.

Studies had to present data about shoulder injuries with sudden or gradual onset. Accepted injury definitions were traumatic dislocation, instability, and subluxation, either self-reported or objectively measured (e.g., medical record or verified diagnosis by a health care professional). Shoulder complaint (e.g., pain) was accepted as a proxy for shoulder injuries.

### Literature search

Systematic literature searches were performed in MEDLINE, CINAHL, EMBASE, and SPORTDiscus from inception to 12th May 2020 and updated 27 February 2021, with no language restrictions. The search strategies were adjusted according to the specifications of the individual database with following key search terms: shoulder joint, hypermobility, and injury. The full search strategy is presented in Additional file 1. Hand-search was performed by screening the references cited in systematic reviews investigating the risk of any sports injury among participants with GJH and shoulder joint hypermobility published within the past 5 years. Lastly, reference lists of the included studies were screened to identify additional studies and forward citation tracking of included studies was performed in Web of Science. All studies identified by the search strategy were imported to EndNote X9 (Clarivate Analytics, Philadelphia, USA).

### Selection of studies

Following the removal of duplicates in EndNote X9, two authors (JRP and JY) independently screened the articles by titles and abstracts in Covidence systematic review software (Veritas Health Innovation, Melbourne, Australia) to identify relevant articles. Full-text articles were then independently screened by two authors (BL and JRP) for inclusion. Disagreements in both title/abstract and full-text screening were resolved by consensus. If unable to reach consensus, a third independent reviewer (JBT) was consulted.

### Data extraction

Data were extracted independently by two authors (BL and JRP) using a standardized data-extraction form including first author, publication year, country, study design, number of participants with and without joint hypermobility, number of participants with and without injury or pain, follow-up time, measure and definition of joint hypermobility, definition of injury, age, sex, % female, BMI, type of activity, weekly sports participation time, sports participation level, and injury outcome measure. Disagreements in data extraction were resolved by consensus. If unable to reach consensus, a third independent reviewer (CBJ) was consulted.

If a study reported more than one injury outcome measure, data was extracted for having shoulder injuries and joint hypermobility compared to those without for the outcome most suitable for this review (i.e., acute shoulder injuries). When studies reported data on both GJH and shoulder joint hypermobility as exposures, data on GJH was extracted. The cut-off points defined by the individual studies to represent athletes with joint hypermobility were extracted. Where studies reported insufficient data to be included in this review, an attempt was made to contact the corresponding author by e-mail.

### Risk of bias assessment of the included studies

Risk of bias was assessed using the Newcastle–Ottawa Scale (NOS) for cohort studies and case-control studies and the modified Newcastle–Ottawa Scale for cross-sectional studies [30], as described in the Cochrane Handbook for Systematic Reviews of Interventions [28]. Two authors (BL and JRP) independently assessed risk of bias of the included studies. Disagreements were resolved by consensus. If unable to reach consensus, a third independent reviewer (CBJ) was consulted. Risk of bias was assessed for three domains: selection of study groups, comparability of the groups, and ascertainment of the exposure and outcome of interest. For cohort studies and case-control studies, eight items were scored with one or two stars, for a maximum total of nine stars representing the lowest risk of bias. For cross-sectional studies, seven items were scored with one or two stars, for a maximum total of 10 stars representing the lowest risk of bias. Thresholds for risk of bias were the following: low, 3 or 4 stars in selection domain and 1 or 2 stars in comparability domain and 2 or 3 stars in outcome/exposure domain; moderate, 2 stars in selection domain and 1 or 2 stars in comparability domain and 2 or 3 stars in outcome/exposure domain; high, 0 or 1 star in selection domain or 0 stars in comparability domain or 0 or 1 star in outcome/exposure domain.

### Overall quality of evidence

The Grading of Recommendations Assessment, Development and Evaluation (GRADE) approach [31] was used to evaluate the overall quality of evidence for the association between joint hypermobility and shoulder injuries, using the GRADEpro Guideline Development Tool (https://gradepro.org/). According to GRADE, observational studies begin as low-quality evidence and can be downgraded to very low based on grading of risk of bias, indirectness, imprecision, inconsistency, and publication bias. Evidence based on observational studies can be upgraded due to dose-response relationship or large effect.

### Strategy for data synthesis

The Odds Ratio (OR) and the corresponding 95% confidence interval (CI) were estimated for each of the included studies. Due to differences among the included studies in participants, sports, and measures used, and therefore an expected heterogeneity, a random-effects meta-analysis was performed to combine the individual study results in Stata IC 16.0 (StataCorp, College Station, Texas, USA). I^2^ statistics were calculated to determine the proportion of variation in the combined estimates due to between-study heterogeneity. Potential publication bias was examined by inspection of forest plots. Subgroup analyses were performed to explore the effect of exposure definition (GJH/shoulder joint hypermobility), level of sport (elite/non-elite), type of activity (sports/military), study type (cohort/cross-sectional and case-control), risk of bias (high/moderate/low), and sex (male, female, mixed-sex). Subgroup analysis on risk of bias was not in the pre-registration. The percentage heterogeneity explained was estimated for each of the above subgroup analyses. Sensitivity analyses were performed using alternative outcomes in studies reporting more than one injury outcome and explored the impact of changing hypermobility measurement tool, where applicable [28]. Further, analyses with and without studies with extreme estimates that conflicted with the rest of the studies were performed as part of the sensitivity analyses.

## Results

### Study selection

Following the initial literature search and after duplicate removal, 6207 records were screened by title and abstract, and 48 full-text articles were considered for inclusion. After review, 42 studies were excluded (Additional file 2) and six studies [4, 22, 32–35] were included in the meta-analysis (study selection process, Additional file 3).

### Study characteristics

The six studies included 2335 (range 118–718) participants (34.1% females) (Table 1). The mean age in four of the individual studies ranged from 18.8 to 23.9 years [4, 22, 32, 35], while one study included athletes between 17 and 37 years [34] and another included collegiate athletes with no age specified [33]. Of the included studies, two were prospective cohort studies [32, 35] with durations of three months [32] and four years [35], one a case-control study [22], and three cross-sectional studies [4, 33, 34]. One study included handball players [4], one gymnasts [33], two athletes from multiple sports [22, 34], and two military personnel [32, 35]. Four studies [22, 32–34] used GJH as exposure (Beighton score, the Hospital Del Mar criteria, or self-reported study-specific questions), and two studies [4, 35] used shoulder joint hypermobility as exposure (clinically verified or self-reported). Shoulder injuries were defined using a variety of outcomes including shoulder injury (acute [34] or traumatic [33]), acute instability, [35] dislocation [22, 32], and pain [4].

**Table 1:** Characteristics of Study Participants, Study Characteristics, and Risk of Bias Assessment of Included Studies

Author, year Country	Study design	Participants, n, age, (female, %)^a^	Sport	Exposure (tool)	Outcome	Risk of bias assessment (NOS)^b^
Azma, 2014 Iran [32]	Prospective cohort (duration 3 months)	Iranian army soldiers, *n* = 718, 19.6 years, (0)	Military	GJH (Beighton score ≥ 4/9)	Shoulder dislocation verified by orthopaedist	9/9, Low
Cameron, 2013 USA [35]	Prospective cohort (duration 4 years)	Freshmen entering the U.S. Military Academy, *n* = 714 (1420 shoulders), 18.8 years, (11.8)	Military	Self-reported history of shoulder instability (previous shoulder dislocation or subluxation) using study-specific questions.	Acute shoulder instability verified by orthopaedic surgeons	8/9, Low
Caplan, 2007 USA [33]	Cross-sectional	Female collegiate gymnasts, *n* = 457, N/A, (100)	Elite gymnastics	GJH (Hyperlaxity signs using a study-specific unvalidated questionnaire, ≥ 2/4)	Traumatic shoulder injury, self-reported using a study-specific questionnaire.	5/10, High
Chahal, 2010 Canada [22]	Case-control	Skeletally mature individuals < 30 years performing recreational or competitive sporting activities, *n* = 149, 23.9 years, (26.2)	Recreational or competitive sports activities	GJH (The Hospital Del Mar, ≥ 4/10 males, ≥ 5/10 females) or Shoulder external rotation exceeding 85°.	Acute, first-time, traumatic anterior shoulder dislocation verified by orthopaedic surgeons	8/9, Low
Myklebust, 2013 Norway [4]	Cross-sectional	Female handball players of the Norwegian elite league, *n* = 179, 22 years, (100)	Elite handball	Anterior shoulder instability (apprehension and relocation tests)	Shoulder pain using a modified Fahlström questionnaire.	6/10, Low
Saremi, 2016 Iran [34]	Cross-sectional	Athletes having a history of sports activity for at least six months, *n* = 118, Range 17–37 years, (32.2)	Professional athletes from multiple sports	GJH (Beighton score ≥ 4/9)	Acute shoulder injury according to documents of local branch of national federation of sports medicine.	6/10, High

*GJH* Generalised Joint Hypermobility, *NOS* Newcastle - Ottawa Quality Assessment Scale with high scores representing low risk of bias. ^a^n = number of participants included in analysis, age reported as mean years unless indicated otherwise

^b^The risk of bias assessment is presented in Additional file 5. Thresholds for risk of bias were the following: low, 3 or 4 stars in selection domain AND 1 or 2 stars in comparability domain AND 2 or 3 stars in outcome/exposure domain; moderate, 2 stars in selection domain AND 1 or 2 stars in comparability domain AND 2 or 3 stars in outcome/exposure domain; high, 0 or 1 star in selection domain OR 0 stars in comparability domain OR 0 or 1 star in outcome/exposure domain

### Association between joint hypermobility and shoulder injuries

**Fig. 1:**
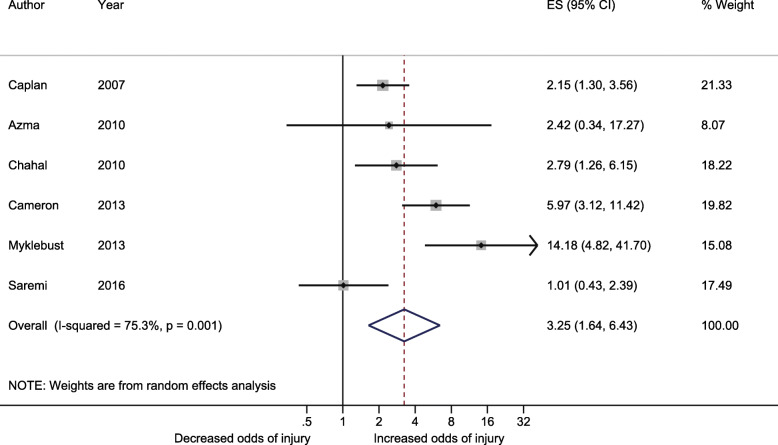
Forest plot showing the association (Odds Ratio (OR) and 95% Confidence Interval (CI)) between joint hypermobility and shoulder injuries (i.e., acute shoulder injuries or activity-related pain) for the six included studies [4, 22, 32–35]

**Fig. 2:**
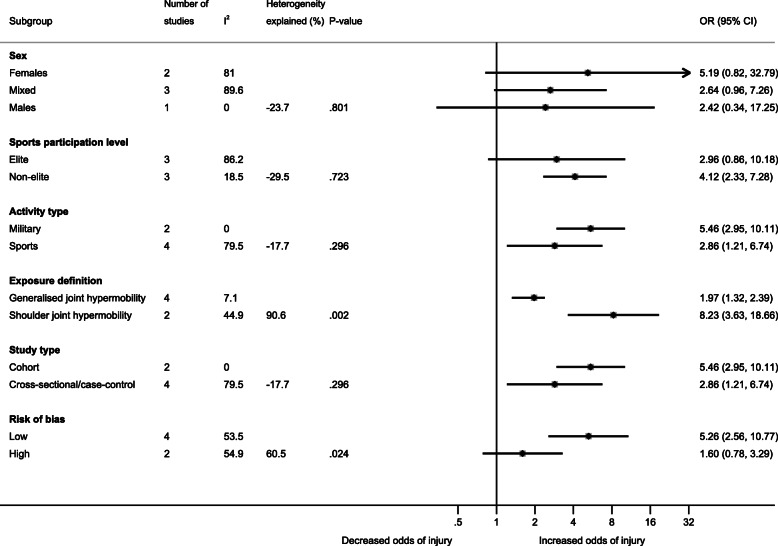
Subgroup analysis showing the association (Odds Ratio (OR) and 95% Confidence Interval (CI)) between joint hypermobility and shoulder injuries (i.e., acute shoulder injuries or activity-related shoulder pain). Heterogeneity explained (%): a positive value denotes less heterogeneity, and a negative value denotes more heterogeneity compared with the primary analysis

Athletes with joint hypermobility were more likely to have shoulder injuries compared with athletes without joint hypermobility (OR = 3.25, 95% CI 1.64, 6.43, I^2^ = 75.3%; *p* = 0.001) (Fig. 1). In the subgroup analyses, there was a significant difference between exposure definitions (GJH, OR = 1.97, 95% CI 1.32, 2.94; shoulder joint hypermobility, OR = 8.23, 95% CI 3.63, 18.66; *p* = 0.002) and risk of bias (low, OR = 5.25, 95% CI 2.56, 10.8; high, OR = 1.6, 95% CI 0.78 to 3.29; *p* = 0.024) (Fig. 2). No significant differences were found in the other subgroup analyses. The exposure definition was the main cause of the substantial heterogeneity observed (90.6% explained). Furthermore, sensitivity analysis excluding two studies with extreme associations [4, 34] resulted in lower heterogeneity without substantially altering the association (OR = 3.17, 95% CI 1.82, 5.53, I^2^ = 50.6%, *p* < 0.001) (Additional File 4). One study [22] used more than one definition of joint hypermobility as exposure, and a sensitivity analysis using the alternative exposure definition (shoulder external rotation instead of the Hospital Del Mar criteria) resulted in a lower association (OR = 3.08, 95% CI 1.55, 6.11, I^2^ = 76.1%, *p* = 0.001) (Additional file 4). One study [34] reported five different shoulder injury-related outcomes, of which “acute shoulder injury” including fractures was chosen for this review. A sensitivity analysis excluding fractures increased the association (OR = 3.54, 95% CI 1.92, 6.53, I^2^ = 67.5%, *p* = 0.009) and reduced the heterogeneity further by 8.5%-points (Additional File 4).

### Risk of bias assessment

Four studies [4, 22, 32, 35] had low risk of bias, and two studies [33, 34] high risk of bias (Additional File 5). For studies with high risk of bias, main reasons included lack of sample size justification and no reporting of the comparability of the participants in the different outcome groups.

### Overall quality of evidence

The level of evidence started at low as we only included observational studies. We downgraded by one level due to the substantial heterogeneity and upgraded by one level because of a strong association [36]. We did not downgrade the quality of evidence based on the risk of bias assessment, as studies with low risk of bias showed the strongest association (Fig. 2). The overall quality of evidence for the estimate was, therefore, judged as low.

## Discussion

Based on data from six studies including 2496 participants, there was a threefold higher odds of having shoulder injuries among athletes with joint hypermobility compared with athletes without joint hypermobility. In the subgroup analysis, using the various GJH definitions from the individual studies resulted in a significantly lower association than using localised shoulder joint hypermobility as exposure. Studies with low risk of bias showed significantly stronger associations compared with high risk of bias. No significant differences were found in the association between subgroups based on type of activity, study type, level of sport, and sex. As the overall quality of evidence was judged as low, the estimates of increased association of having shoulder injuries in patients with joint hypermobility must be interpreted with caution.

Our results are comparable with previously reported associations between GJH and knee joint injury in athletes, [26] in the general population, [27] and the association for the presence of GJH in patients with an anterior cruciate ligament injury [25]. In contrast, there was no association between joint hypermobility and ankle joint injury, [26] suggesting that the association may be joint specific. One study [37] that was excluded in this review due to the low mean age of participants reported that adolescent swimmers with increased external rotation range of motion of more than 100° also had higher risk of developing a shoulder injury, supporting our findings.

Various definitions of joint hypermobility have been used to investigate its association with shoulder injuries. The Beighton score is currently used in most epidemiological studies and consists of nine dichotomous joint hypermobility tests, where a tested joint is either hypermobile (score = 1) or not hypermobile (score = 0), with the scores ranging from 0 and 9, and higher scores indicating more joints with joint hypermobility/hyperlaxity [38, 39]. The Beighton score with a cut-off of ≥4/9, as previously recommended to classify GJH in adults [40], was used by two studies [22, 32]. A general limitation of using the Beighton score in shoulder studies is that the shoulder joint is not included in the test battery. However, being classified with GJH by the Beighton score builds on the assumption that all joints, including the shoulder, are hypermobile. In contrast, the Hospital Del Mar criteria (cut-off 4 for males or 5 for females out of 10), as used by one study [22], includes the shoulder external rotation test >85° in neutral position to classify GJH. The association for shoulder injury varied significantly with exposure definition between GJH (OR = 1.97) and localised shoulder joint hypermobility (OR = 8.23). Another contributing factor is that local shoulder hypermobility may not be captured when using common tests for GJH (e.g., Beighton score). It therefore seems important to include shoulder-specific measures of joint hypermobility, such as a positive apprehension test for anterior instability [4], the shoulder external rotation test >85° [22], or self-reported previous shoulder instability [35], when assessing the risk of shoulder injuries in athletes with joint hypermobility.

There was substantial heterogeneity in the primary meta-analysis, mainly due to the exposure definition and the risk of bias (Fig. 2). Furthermore, the I^2^ value was reduced substantially from 75.3 to 50.6% when excluding two studies with the most extreme estimates, [4, 34] of which Myklebust et al. [4] reported the strongest association among the included studies between shoulder laxity (anterior instability tests) and shoulder pain in female elite handball players. This may be explained by the population (only female athletes), outcome (shoulder pain), exposure definition, and/or the specific sport (e.g. that shoulder pain is very common in handball) [4]. Handball players with joint hypermobility may be more exposed to shoulder pain. The study by Saremi et al. [34] showed the weakest association with shoulder injuries and was the only one in the meta-analysis including fractures, which were more prevalent in athletes without joint hypermobility. None of the sensitivity analyses showed important changes in the reported associations.

To date, little is known about the underlying pathophysiological mechanism of joint hypermobility [18]. However, research suggests that impaired collagen synthesis results in laxity of the connective tissue matrix and affects the stability of the joint capsules and the extensibility of ligaments, tendons, and the skin, thereby increasing the demands on the active muscular stabilisers [41]. In sports where high flexibility is required such as swimming, ballet, or dancing, joint hypermobility is often considered to be advantageous [42–46]. On the contrary, it may be a disadvantage being an athlete with joint hypermobility, as this condition may increase the susceptibility for sports-related injuries, [26, 27, 44, 47–50] potentially caused by inherent strength deficits, increased muscular fatigue, and/or poor joint stability [20, 51]. Although there are some older studies showing a similar or even reduced injury risk for athletes with GJH, [52, 53] recent studies support joint hypermobility as a potentially important factor in injury aetiology [26, 27].

### Limitations

The six studies included in this systematic review and meta-analysis varied in terms of study type, exposure, and outcome definitions/criteria (e.g., including measures of pain), resulting in large heterogeneity. For example, in two studies [33, 35] the exposure was self-reported without any clinical verification of the condition. However, the large heterogeneity was primarily explained by exposure definition and risk of bias, and the completed sensitivity analyses generally yielded similar results. Since shoulder pain can be related to both joint hypermobility and shoulder injury, we performed a post hoc sensitivity analysis excluding the paper by Myklebyst et al. [4] and found that it did not substantially change the estimate (OR 2.52, 95% CI 1.36, 4.66). Aetiology of shoulder injuries among athletes is known to be multifactorial, but only one of the included studies [35] used multivariable analyses to identify the combination of risk factors associated with shoulder injury [54]. As one of the included studies was a case-control design, the OR was presented in the meta-analysis. Considering the prevalence range of the outcome in the non-hypermobile population (0.4 to 32.8%), estimated relative risks are 2.65, 2.24, or 1,94 (reference prevalence of 10, 20, 30%, respectively). Therefore, the reported association measured as OR may overestimate the associations between joint hypermobility and shoulder injuries. Due to the few studies and low quality of evidence, future research is very likely to change the estimated strength of this effect.

### Perspective

Due to the increased odds of having shoulder injuries in athletes with joint hypermobility, the current findings highlight the need to focus on prevention of shoulder injuries in athletes with joint hypermobility and the subsequent tailored treatment after shoulder injury. Future prospective cohort studies on risk factors for sustaining shoulder injuries should include joint hypermobility as a potential variable using high-quality design and standardised testing methods. More studies are needed to compare non-contact with contact sports, level of sports participation, and sex differences, which may be important for establishing injury risk in sport for both GJH and localised shoulder joint hypermobility [55]. We also suggest future risk factor studies to assess whether joint hypermobility results in greater time-loss, as no data is currently available about the severity and consequences of shoulder injuries in this population. This knowledge is important to target future treatment and prevention programmes more specifically.

## Conclusions

There was a threefold higher odds of having shoulder injuries among athletes with joint hypermobility compared with athletes without joint hypermobility. This finding highlights the need for prevention of shoulder injuries in athletes with joint hypermobility. However, the overall quality of evidence was low, meaning that future research is very likely to change the estimated strength of the association.

## Supplementary Information

**Additional file 1.** Search matrix.

**Additional file 2.** Excluded full-text articles.

**Additional file 3.** Study selection process.

**Additional file 4.** Sensitivity analyses excluding extreme results, using different exposure definitions, and excluding shoulder fractures.

**Additional file 5.** Risk of bias assessment.

## Data Availability

The datasets used and analysed during the current study are available from the corresponding author on request.

## Abbreviations

CI: Confidence interval
GJH: Generalised joint hypermobility
GRADE: Grading of Recommendations Assessment, Development and Evaluation
OR: Odds Ratio
PRISMA: Preferred Reporting Items for Systematic Reviews and Meta-analysis guidelines
