# Supplementary material for: Joint hypermobility in athletes is associated with shoulder injuries: a systematic review and meta-analysis
Source: BMC Musculoskelet Disord. 2021 Apr 26;22:389. doi: 10.1186/s12891-021-04249-x (PMC8077913; doi:10.1186/s12891-021-04249-x)
Supplement: Supplementary file 1 — Additional file 1. Search matrix. [file 12891_2021_4249_MOESM1_ESM.docx]

A Boolean search strategy was employed, including subject headings, text words (including alternative spelling), syntax (truncation, proximity operations etc), searching in the appropriate fields and correct use of And/Or. We adjusted the search strategies according to the specifications of the individual database with the following key search terms: shoulder joint, hypermobility, and injury.

Search strategy updated 27 February 2021.

Ovid MEDLINE(R) ALL <1946 to February 26, 2021>

1 Shoulder Pain/ 5022

2 Shoulder Fractures/ 3422

3 Shoulder Dislocation/ 5947

4 Shoulder Impingement Syndrome/ 1801

5 Rotator cuff injuries/ 6114

6 Shoulder injuries/ 2050

7 ((Athletic injur* or sports injur*) and (shoulder or glenohumeral or gleno-humeral or rotator cuff or acromioclavicular or scapula*)).mp. 2555

8 ((shoulder or glenohumeral or gleno-humeral or rotator cuff or acromioclavicular or scapula) adj3 (injur* or problem* or pain* or instab* or strain* or fracture* or dislocation* or syndrom* or pathology or subluxation* or impingement)).mp. 34983

9 1 or 2 or 3 or 4 or 5 or 6 or 7 or 8 35718

10 hypermob*.mp. 3514

11 Beighton*.mp. 452

12 (hyperlax* or lax*).mp. 19042

13 Joint instability/ or joint instab*.mp. 21895

14 10 or 11 or 12 or 13 40200

15 9 and 14 **3640**

Embase Classic+Embase <1947 to 2021 February 26>

1 shoulder pain/ 17540

2 shoulder fracture/ 1345

3 shoulder dislocation/ 6603

4 shoulder impingement syndrome/ 2947

5 rotator cuff injury/ 2690

6 shoulder injury/ 4666

7 rotator cuff rupture/ 7589

8 ((Athletic injur* or sports injur*) and (shoulder or glenohumeral or gleno-humeral or rotator cuff or acromioclavicular or scapula*)).mp. 664

9 ((shoulder or glenohumeral or gleno-humeral or rotator cuff or acromioclavicular or scapula) adj3 (injur* or problem* or pain* or strain* or fracture* or dislocation* or syndrom* or pathology or subluxation* or impingement)).mp. 46649

10 1 or 2 or 3 or 4 or 5 or 6 or 7 or 8 or 9 50885

11 joint instability/ or joint instab*.mp. 13019

12 joint hypermobility/ or joint laxity/ 5911

13 hypermob*.mp. 5910

14 Beighton*.mp. 837

15 (hyperlax* or lax*).mp. 38745

16 11 or 12 or 13 or 14 or 15 54243

17 10 and 16 **2210**

**Print Search History 27-02-2021**

| 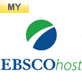 | Saturday, February 27, 2021 8:37:50 AM |
| --- | --- |

| **#** | **Query** | **Results** |
| --- | --- | --- |
| S19 | S13 AND S18 | **2,422** |
| S18 | S14 OR S15 OR S16 OR S17 | 16,177 |
| S17 | hyperlax* OR lax* | 6,982 |
| S16 | Beighton* | 301 |
| S15 | hypermob* | 1,211 |
| S14 | (MH "Joint Instability") OR "joint instability" | 9,871 |
| S13 | S1 OR S2 OR S3 OR S4 OR S5 OR S6 OR S7 OR S8 OR S10 OR S12 | 45,307 |
| S12 | (S9) and (S11) | 27,498 |
| S11 | injur* or problem* or pain* or instab* or strain* or fracture* or dislocation* or syndrom* or pathology or subluxation* or impingement | 1,545,537 |
| S10 | S8 and S9 | 1,763 |
| S9 | shoulder or glenohumeral or gleno-humeral or rotator cuff or acromioclavicular or scapula* | 37,615 |
| S8 | (MH "Athletic Injuries") OR "Athletic Injur*" OR "sport* injur*" | 19,530 |
| S7 | (MH "Rotator Cuff Injuries") | 2,995 |
| S6 | (MH "Shoulder Injuries") | 2,229 |
| S5 | (MH "Shoulder Impingement Syndrome") | 1,382 |
| S4 | (MH "Shoulder Dislocation") | 1,609 |
| S3 | (MH "Shoulder Labrum Tear") | 174 |
| S2 | (MH "Shoulder Fractures") | 1,142 |
| S1 | (MH "Shoulder Pain") | 4,148 |

**Database: SPORTDiscus**

Search Strategy:

--------------------------------------------------------------------------------

S3) DE ”SHOULDER pain” OR DE ”SHOULDER joint injuries” OR ( ((athletic injur* OR sports injur*) AND (shoulder OR glenohumeral OR rotator cuff or acromioclavicular or scapula*)) ) OR ( ((shoulder or glenohumeral or rotator cuff or acromioclavicular or scapula*) AND (injur* or problem or pain or strain or fracture or dislocation or syndrom or pathology or subluxation* or impingement)) ) AND (DE “HYPERMOBILITY of joints” OR Hypermob* OR Beighton* OR ( (hyperlax or lax*) ) ) **1028**

S2) DE “HYPERMOBILITY of joints” OR Hypermob* OR Beighton* OR ( (hyperlax or lax*) )

6674

S1) DE ”SHOULDER pain” OR DE ”SHOULDER joint injuries” OR ( ((athletic injur* OR sports injur*) AND (shoulder OR glenohumeral OR rotator cuff or acromioclavicular or scapula*)) ) OR ( ((shoulder or glenohumeral or rotator cuff or acromioclavicular or scapula*) AND (injur* or problem or pain or strain or fracture or dislocation or syndrom or pathology or subluxation* or impingement)) )

15713
