# Supplementary material for: Joint hypermobility in athletes is associated with shoulder injuries: a systematic review and meta-analysis
Source: BMC Musculoskelet Disord. 2021 Apr 26;22:389. doi: 10.1186/s12891-021-04249-x (PMC8077913; doi:10.1186/s12891-021-04249-x)
Supplement: Supplementary file 2 — Additional file 2. Excluded full-text articles. [file 12891_2021_4249_MOESM2_ESM.docx]

**Additional file 2**List of excluded full-text articles

Of the 48 full-text articles identified for potential inclusion into the review, 42 were excluded.

**Exposure not defined**

Borsa PA, Scibek JS, Jacobson JA, Meister K. Sonographic stress measurement of glenohumeral joint laxity in collegiate swimmers and age-matched controls. American Journal of Sports Medicine.

2005;33(7):1077-84.

Greipp JF. Swimmer's Shoulder: The Influence of Flexibility and Weight Training. Phys Sportsmed. 1985;13(8):92-105.

Harrington S, Meisel C, Tate A. A cross-sectional study examining shoulder pain and disability in Division I female swimmers. J Sport Rehabil. 2014;23(1):65-75.

Lippincott EL. Predictors of Shoulder Injuries in Female Collegiate Swimmers. Predictors of Shoulder Injuries in Female Collegiate Swimmers. 2018:1-.

McMaster WC, Roberts A, Stoddard T. A correlation between shoulder laxity and interfering pain in competitive swimmers. / Une correlation entre une laxite au niveau de l'epaule et la douleur s'y rapportant aupres de nageurs faisant de la competition. American Journal of Sports Medicine. 1998;26(1):83-6.

Ozcaldiran B. A relation between static flexibility and shoulder pain in competitive age-group swimmers. The Pain Clinic. 2002;14(2):159-63.

Tate A, Turner GN, Knab SE, Jorgensen C, Strittmatter A, Michener LA. Risk factors associated with shoulder pain and disability across the lifespan of competitive swimmers. Journal of athletic training. 2012;47(2):149-58.

Warner JJP, Micheli LJ, Arslanian LE, Kennedy J, Kennedy R. Patterns of flexibility, laxity, and strength in normal shoulders and shoulders with instability and impingement. American Journal of Sports Medicine. 1990;18(4):366-75.

**Wrong outcome**

Armstrong R. The Beighton Score and Injury in Dancers: A Prospective Cohort Study. J Sport Rehabil. 2019 Oct 18;29(5):563-571.

Armstrong R, Greig M. The Beighton score as a predictor of Brighton criteria in sport and dance. Physical Therapy in Sport. 2018;32:145-54.

Bak K, Magnusson SP. Shoulder strength and range of motion in symptomatic and pain-free elite swimmers. Am J Sports Med. 1997;25(4):454-9.

Bansal S, Sinha AGK, Sandhu JS. Shoulder impingement syndrome among competitive swimmers in India - prevalence, evaluation and risk factors. Journal of Exercise Science & Fitness. 2007;5(2):102-8.

Beach ML, Whitney SL, Dickoff-Hoffman S. Relationship of shoulder flexibility, strength, and endurance to shoulder pain in competitive swimmers. J Orthop Sports Phys Ther. 1992;16(6):262-8.

Bin Abd Razak HR, Bin Ali N, Howe TS. Generalized ligamentous laxity may be a predisposing factor for musculoskeletal injuries. J Sci Med Sport. 2014;17(5):474-8.

Bohu Y, Klouche S, Lefevre N, Peyrin JC, Dusfour B, Hager JP, Ribaut A, Herman S. The epidemiology of 1345 shoulder dislocations and subluxations in French Rugby Union players: a five-season prospective study from 2008 to 2013. Br J Sports Med. 2015 Dec;49(23):1535-40.

Bronner S, Bauer NG. Risk factors for musculoskeletal injury in elite pre-professional modern dancers: A prospective cohort prognostic study. Phys Ther Sport. 2018 May;31:42-51.

Bukva B, VrgoČ G, MadiĆ DM, SporiŠ G, TrajkoviĆ N. Correlation between hypermobility score and injury rate in artistic gymnastics. Journal of Sports Medicine & Physical Fitness. 2019;59(2):330-40.

Decoster LC, Bernier JN, Lindsay RH, Vailas JC. Generalized Joint Hypermobility and Its Relationship to Injury Patterns Among NCAA Lacrosse Players. J Athl Train. 1999;34(2):99-105.

Gohlke F, Lippert MJ, Keck O. [Instability and impingement of the shoulder of the high performance athlete in overhead stress]. Sportverletz Sportschaden. 1993;7(3):115-21.

Goodman AD, DeFroda SF, Gil JA, Kleiner JE, Li NY, Owens BD. Season-Ending Shoulder Injuries in the National Collegiate Athletic Association: Data From the NCAA Injury Surveillance Program, 2009-2010 Through 2013-2014. American Journal of Sports Medicine. 2018;46(8):1936-42.

Konopinski MD, Jones GJ, Johnson MI. The effect of hypermobility on the incidence of injuries in elite-level professional soccer players: a cohort study. Am J Sports Med. 2012 Apr;40(4):763-9.

Nunes NM, Haddad JJ, Bartlett DJ, Obright KD. Musculoskeletal injuries among young, recreational, female dancers before and after dancing in pointe shoes. Pediatr Phys Ther. 2002 Summer;14(2):100-6.

Owens BD, Campbell SE, Cameron KL. Risk Factors for Posterior Shoulder Instability in Young Athletes. American Journal of Sports Medicine. 2013;41(11):2645-9.

Radlinska N, Bac A, Zawada K, Zalewski M, Wozniacka R. PREVALENCE OF GENERALISED JOINT HYPERMOBILITY IN RELATION TO SELECTED MEDICAL AND TRAINING INDICATORS IN SWIMMERS: RANDOMISED CONTROL STUDY. South African Journal for Research in Sport Physical Education and Recreation. 2019;41(3):75-86.

Ruemper A, Watkins K. Correlations Between General Joint Hypermobility and Joint Hypermobility Syndrome and Injury in Contemporary Dance Students. J Dance Med Sci. 2012 Dec;16(4):161-6.

Rupp S, Berninger K, Hopf T. Shoulder problems in high level swimmers--impingement, anterior instability, muscular imbalance? Int J Sports Med. 1995;16(8):557-62.

Scheper MC, de Vries JE, de Vos R, Verbunt J, Nollet F, Engelbert RH. Generalized joint hypermobility in professional dancers: a sign of talent or vulnerability? Rheumatology (Oxford). 2013 Apr;52(4):651-8.

Steele VA, White JA. Injury prediction in female gymnasts. British Journal of Sports Medicine. 1986;20(1):31-3.

Sugimoto D, Loiacono AJ, Blenis A, Morse JM, Borg DR, Meehan WP 3rd. Risk Factors in Elite, Adolescent Male Soccer Players: Prospective Study. Clin Pediatr (Phila). 2020 Jun;59(6):596-605.

Sueyoshi T, Emoto G, Yuasa T. Generalized Joint Laxity and Ligament Injuries in High School-Aged Female Volleyball Players in Japan. Orthopaedic Journal of Sports Medicine. 2016;4(10).

**Wrong study design**

Hill L, Collins M, Posthumus M. Risk factors for shoulder pain and injury in swimmers: A critical systematic review. Physician and Sportsmedicine. 2015;43(3):412-20.

**Wrong population**

Cameron KL, Duffey ML, DeBerardino TM, Stoneman PD, Jones CJ, Owens BD. Association of generalized joint hypermobility with a history of glenohumeral joint instability. J Athl Train. 2010;45(3):253-258.

de Oliveira VMA, Pitangui ACR, Gomes MRA, da Silva HA, dos Passos MHP, de Araujo RC. Shoulder pain in adolescent athletes: prevalence, associated factors and its influence on upper limb function. Brazilian Journal of Physical Therapy. 2017;21(2):107-13.

Jacobson RP, Benson CJ. Amateur volleyball attackers competing despite shoulder pain: analysis of play habits, anthropometric data, and specific pathologies. Physical Therapy in Sport. 2001;2(3):112-22.

Leppanen M, Pasanen K, Kannus P, Vasankari T, Kujala UM, Heinonen A, Parkkari J. Epidemiology of Overuse Injuries in Youth Team Sports: A 3-year Prospective Study. Int J Sports Med. 2017;38(11):847-56.

Pasque CB, Hewett TE. A prospective study of high school wrestling injuries. American Journal of Sports Medicine. 2000;28(4):509-15.

Ranalletta M, Bongiovanni S, Suarez F, Ovenza JML, Maignon G. Do patients with traumatic recurrent anterior shoulder instability have generalized joint laxity? Clinical Orthopaedics and Related Research. 2012;470(4):957-60.

Sein ML, Walton J, Linklater J, Appleyard R, Kirkbride B, Kuah D, Murrell GA. Shoulder pain in elite swimmers: primarily due to swim-volume-induced supraspinatus tendinopathy. British Journal of Sports Medicine. 2010;44(2):105-13.

Walker H, Gabbe B, Wajswelner H, Blanch P, Bennell K. Shoulder pain in swimmers: A 12-month prospective cohort study of incidence and risk factors. Physical Therapy in Sport. 2012;13(4):243-9.

**Language**

Kawahara K, Chosa E, Yamamoto K, Tajima T, Sonoda N, Tajima N. Relationship between athletic disability, injury and joint laxity, muscle tightness for young athletes of medical check-up in Miyazaki. Japanese Journal of Clinical Sports Medicine. 2010;18(1):59-66.

**Abstract**

Muhammad AA, Jenkins P, Ashton F, Christopher MR. HYPERMOBILITY- A RISK FACTOR FOR RECURRENT SHOULDER DISLOCATIONS. British Journal of Sports Medicine. 2013;47(10):4-5.

Sein M, Walton J, Linklater J, Appleyard R, Kirkbride B, Kuah D, Murrell G. Shoulder laxity and impingement in elite swimmers. (Abstract). Journal of Science & Medicine in Sport. 2005;8(4 Supplement)
