# Supplementary material for: Joint hypermobility in athletes is associated with shoulder injuries: a systematic review and meta-analysis
Source: BMC Musculoskelet Disord. 2021 Apr 26;22:389. doi: 10.1186/s12891-021-04249-x (PMC8077913; doi:10.1186/s12891-021-04249-x)
Supplement: Supplementary file 3 — Additional file 3. Study selection process. [file 12891_2021_4249_MOESM3_ESM.docx]

**Additional file 3. Study selection process**

6207

R

ecords identified through

database searching

n

=

(

9300

)

**Screening**

**Included**

**Eligibility**

**Identification**

A

dditional

records identified

through other sources

(

n =

4

8

)

Records

after duplicates removed

(

n =

6207

)

Records

screened

(

n =

)

R

ecords exc

l

uded

(

n

=

6159

)

F

ull

-

text articles assessed

for eligibility

(

n =

4

8

)

F

ull

-

text articles excluded,

with reasons

(

n =

42

)

Exposure not defined (n = 8)

Wrong outcome (n = 22)

W

rong study

desig

n

(

n =

1)

Wrong population (n =

8

)

Language (

n = 1)

Abstrac

t (

n = 2)

S

tudies included in

qualitative synthesis

(

n =

6

)

S

tudies included in

quantitative synthesis

(

meta

-

analysis)

(

n = 6

6

)
