# Supplementary material for: Joint hypermobility in athletes is associated with shoulder injuries: a systematic review and meta-analysis
Source: BMC Musculoskelet Disord. 2021 Apr 26;22:389. doi: 10.1186/s12891-021-04249-x (PMC8077913; doi:10.1186/s12891-021-04249-x)
Supplement: Supplementary file 4 — Additional file 4. Sensitivity analyses excluding extreme results, using different exposure definitions, and excluding shoulder fractures. [file 12891_2021_4249_MOESM4_ESM.docx]

**Additional file 4**

Sensitivity analyses

Additional file figure 1:

The substantial heterogeneity was partially explained by two studies with extreme estimates^1 2^, and excluding these studies in a sensitivity analysis resulted in moderate heterogeneity without substantially altering the association (Odds Ratio 3.17, 95% Confidence Interval 1.82 to 5.53, I^2^=50.6%).
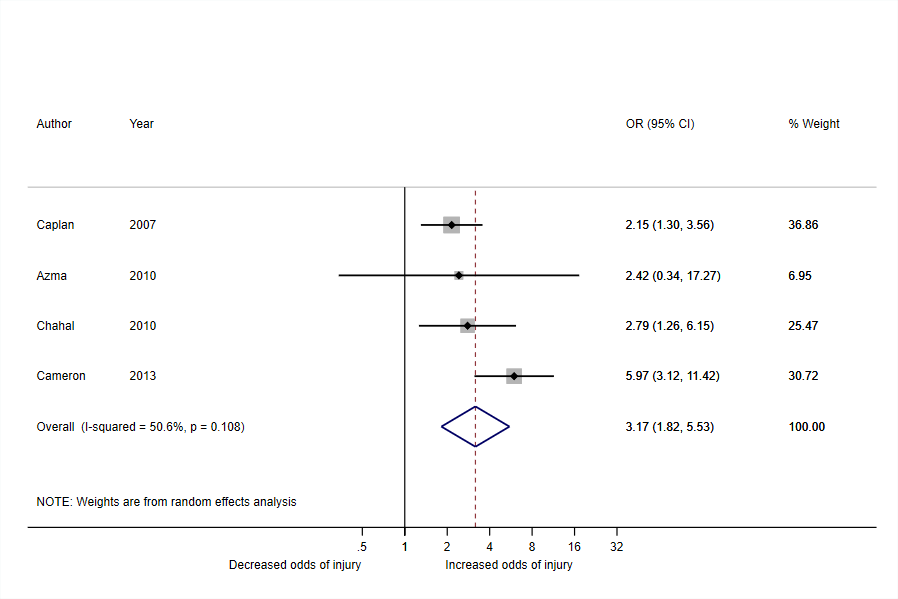


Additional file figure 2: one study^3^ used more than one definition of joint hypermobility as exposures, and a sensitivity analysis using data from the exposure considered less relevant for our objective resulted in a weaker association (Odds Ratio = 3.08, 95% Confidence Interval 1.55 to 6.11, I^2^ = 76.1%).


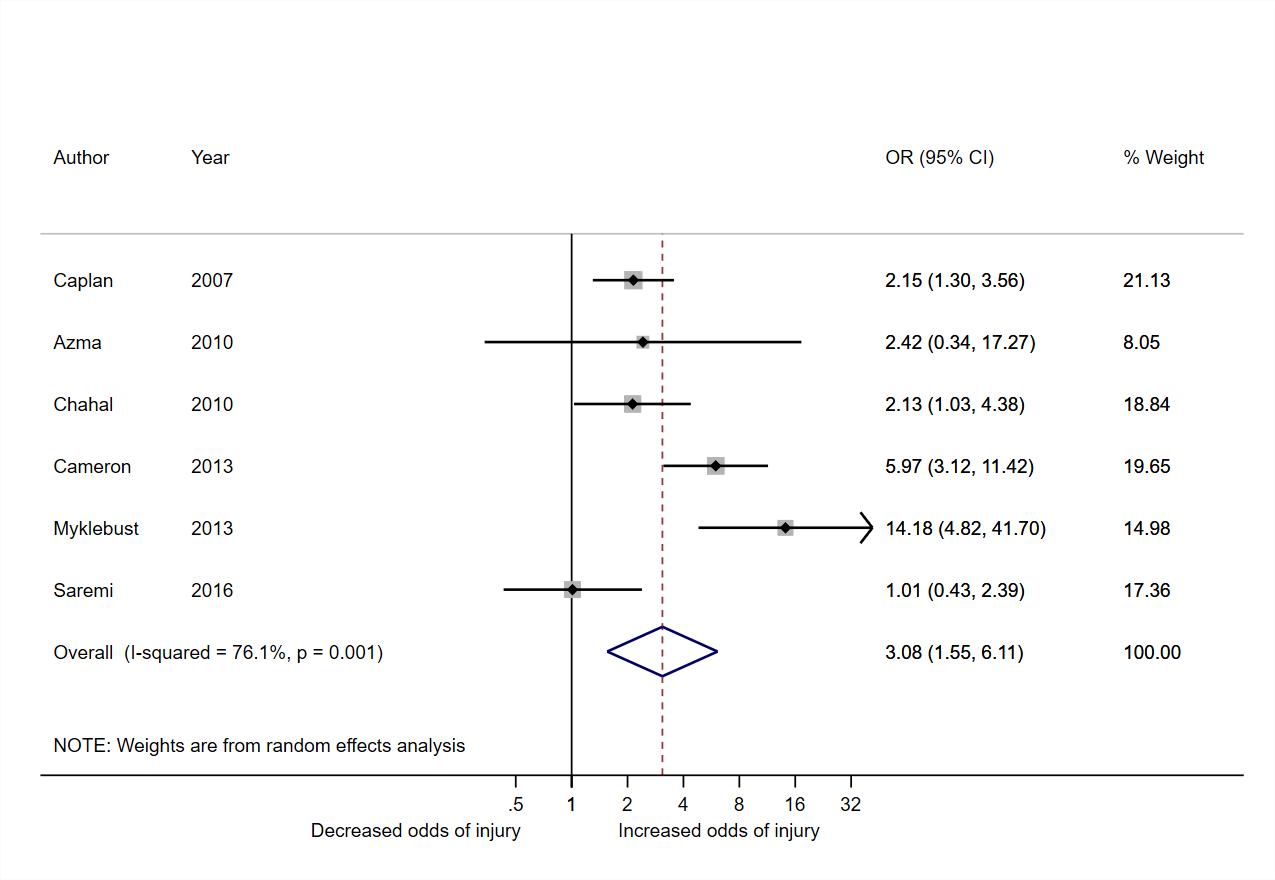


Additional file figure 3: one study^1^ did not show an increased odds ratio for having shoulder injury (Odds Ratio 1.01, 95% Confidence Interval 0.43 to 2.39). Since this was the only study in the meta-analysis including fractures as an outcome, a sensitivity analysis was performed without fractures, which resulted in a stronger association (Odds Ratio = 3.54, 95% Confidence Interval 1.92 to 6.53, I^2^ = 67.5%).


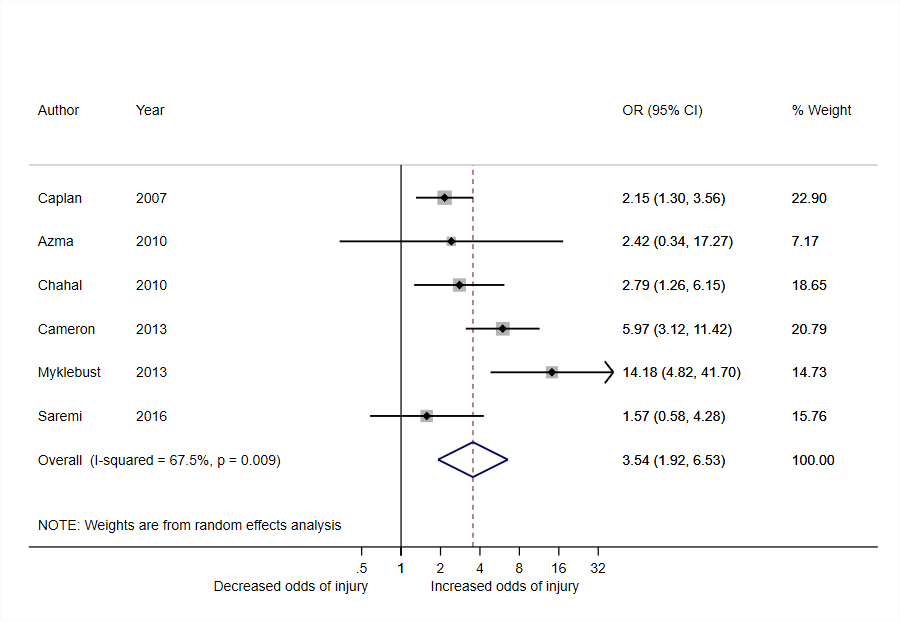


References

1. Saremi H, Yavarikia A, Jafari N. Generalized ligamentous laxity: An important predisposing factor for shoulder injuries in athletes. *Iranian Red Crescent Medical Journal* 2016;18 (6) (no pagination)(e38903) doi: <http://dx.doi.org/10.5812/ircmj.38903>

2. Myklebust G, Hasslan L, Bahr R, et al. High prevalence of shoulder pain among elite Norwegian female handball players. *Scandinavian Journal of Medicine and Science in Sports* 2013;23(3):288-94. doi: <http://dx.doi.org/10.1111/j.1600-0838.2011.01398.x>

3. Chahal J, Leiter J, McKee MD, et al. Generalized ligamentous laxity as a predisposing factor for primary traumatic anterior shoulder dislocation. *Journal of Shoulder and Elbow Surgery* 2010;19(8):1238-42. doi: <http://dx.doi.org/10.1016/j.jse.2010.02.005>
