# Supplementary material for: Joint hypermobility in athletes is associated with shoulder injuries: a systematic review and meta-analysis
Source: BMC Musculoskelet Disord. 2021 Apr 26;22:389. doi: 10.1186/s12891-021-04249-x (PMC8077913; doi:10.1186/s12891-021-04249-x)
Supplement: Supplementary file 5 — Additional file 5. Risk of bias assessment. [file 12891_2021_4249_MOESM5_ESM.docx]

| **Additional file 5. Table. The Newcastle-Ottawa Scale (NOS) quality assessment of the included studies in this meta-analysis** | | | | | | | | |  |
| --- | --- | --- | --- | --- | --- | --- | --- | --- | --- |
| Cohort studies | | | | | | | | |  |
| **Author, year^reference^** | **Selection of cohorts** | | | | **Comparability of cohorts** | **Outcome** | | | **Overall risk of bias^a^** |
|  | Representativeness of the exposed cohort | Selection of the non-exposed cohort | Ascertainment of exposure | Demonstration that outcome of interest was not present at start of study | Comparability of cohorts on the basis of the design or analysis | Assessment of outcome | Was follow up long enough for outcomes to occur | Adequacy of follow up of cohorts |  |
| Azma, 2014^1^ | ☆ | ☆ | ☆ | ☆ | ☆☆ | ☆ | ☆ | ☆ | Low |
| Cameron, 2013^2^ | ☆ | ☆ |  | ☆ | ☆☆ | ☆ | ☆ | ☆ | Low |
|  |  |  |  |  |  |  |  |  |  |
| Case-control studies | | | | | | | | |  |
| **Author, year^reference^** | **Selection of case and controls** | | | | **Comparability of cases and controls** | **Exposure** | | | **Overall risk of bias^a^** |
|  | Is the case definition adequate | Representativeness of the cases | Selection of Controls | Definition of Controls | Comparability of cases and controls on the basis of the design or analysis | Ascertainment of Exposure | Same method of ascertainment for cases and controls | Non-Response Rate |  |
| Chahal, 2010^3^ | ☆ | ☆ | ☆ | ☆ | ☆☆ |  | ☆ | ☆ | Low |
|  |  |  |  |  |  |  |  |  |  |
| Cross-sectional studies | | | | | | | | |  |
| **Author, year^reference^** | **Selection** | | | | **Comparability** | **Outcome** | | | **Overall risk of bias^a^** |
|  | Representativeness of the sample | Sample size | Non-respondents | Ascertainment of the exposure (risk factor) | The subjects in different outcome groups are comparable, based on the study design or analysis. | Assessment of the outcome | Statistical test |  |  |
| Caplan, 2007^4^ | ☆ |  | ☆ | ☆ |  | ☆ | ☆ |  | High |
| Myklebust, 2013^5^ | ☆ |  |  | ☆☆ | ☆ | ☆ | ☆ |  | Low |
| Saremi, 2016^6^ | ☆ | ☆ |  | ☆☆ |  | ☆ | ☆ |  | High |

^a^Thresholds for risk of bias were the following: low, 3 or 4 stars in selection domain AND 1 or 2 stars in comparability domain AND 2 or 3 stars in outcome/exposure domain; moderate, 2 stars in selection domain AND 1 or 2 stars in comparability domain AND 2 or 3 stars in outcome/exposure domain; high, 0 or 1 star in selection domain OR 0 stars in comparability domain OR 0 or 1 stars in outcome/exposure domain.

**References**

1. Azma K, Mottaghi P, Hosseini A, et al. Benign joint hypermobility syndrome in soldiers; what is the effect of military training courses on associated joint instabilities? *J Res Med Sci* 2014;19(7):639-43.

2. Cameron KL, Mountcastle SB, Nelson BJ, et al. History of shoulder instability and subsequent injury during four years of follow-up: a survival analysis. *Journal of Bone & Joint Surgery, American Volume* 2013;95(5):439-45. doi: 10.2106/JBJS.L.00252

3. Chahal J, Leiter J, McKee MD, et al. Generalized ligamentous laxity as a predisposing factor for primary traumatic anterior shoulder dislocation. *Journal of Shoulder and Elbow Surgery* 2010;19(8):1238-42. doi: <http://dx.doi.org/10.1016/j.jse.2010.02.005>

4. Caplan J, Julien TP, Michelson J, et al. Multidirectional instability of the shoulder in elite female gymnasts. *American journal of orthopedics (Belle Mead, NJ)* 2007;36(12):660-65.

5. Myklebust G, Hasslan L, Bahr R, et al. High prevalence of shoulder pain among elite Norwegian female handball players. *Scandinavian Journal of Medicine and Science in Sports* 2013;23(3):288-94. doi: <http://dx.doi.org/10.1111/j.1600-0838.2011.01398.x>

6. Saremi H, Yavarikia A, Jafari N. Generalized ligamentous laxity: An important predisposing factor for shoulder injuries in athletes. *Iranian Red Crescent Medical Journal* 2016;18 (6) (no pagination)(e38903) doi: <http://dx.doi.org/10.5812/ircmj.38903>
